# Irritable Bowel Syndrome in a Bangladeshi Urban Community: Prevalence and Health Care Seeking Pattern

**DOI:** 10.4103/1319-3767.56099

**Published:** 2009-10

**Authors:** Irin Perveen, Mahmud Hasan, Mohammed A. Masud, Mohammed M. R. Bhuiyan, Mohammed M. Rahman

**Affiliations:** Department of Gastroenterology, BIHS Hospital, Dhaka, Bangladesh; 1 Department of Gastroenterology, Bangabandhu Sheikh Mujib Medical University, Dhaka, Bangladesh; 2 Department of Gastroenterology, Dhaka Medical College Hospital, Dhaka, Bangladesh; 3 Department of Gastroenterology, Bangubandhu Sheikh Mujib Medical University, Dhaka, Bangladesh

**Keywords:** Healthcare-seeking, irritable bowel syndrome prevalence, Rome-II, urban Bangladesh

## Abstract

**Background/Aims:**

Although irritable bowel syndrome (IBS) is a common gastrointestinal disorder, its prevalence is unknown, especially in the urban population of Bangladesh. This community-based study aimed to find out the prevalence of IBS and healthcare-seeking patterns using the Rome-II definition.

**Materials and Methods:**

A population-based cross-sectional survey of 1503 persons aged 15 years and above was carried out in an urban community of Bangladesh. The subjects were interviewed using a valid questionnaire based on Rome-II criteria in a home setting. Statistical analysis was performed with Statistical Package for Social Science (SPSS) Programmers and the level of significance was set at *P* ≤ 0.05.

**Results:**

A response rate of 97.2% yielded 1503 questionnaires for analysis. The prevalence of IBS was found to be 7.7% (*n* = 116) with a male to female ratio of 1:1.36 (49 *vs*. 67). “Diarrhoea-predominant IBS” (50%, *n* = 58) was the predominant IBS subgroup. Symptoms of abdominal pain associated with a change in stool frequency (100%) and consistency (88.8%) were quite common. All IBS symptoms were more prevalent among women (*P* < 0.000). In the past one year, 65.5% (*n* = 76) IBS subjects had consulted a physician with a slightly higher rate of women consulters (68.6 *vs.* 61.2%). The main predictor for healthcare-seeking was the presence of multiple dyspeptic symptoms.

**Conclusions:**

The prevalence of IBS in the urban community was found to be similar to that in rural communities. A higher rate of consultation was found among urban IBS subjects than in the rural subjects, with sex not seen to be a discriminator to seek consultation.

The overall prevalence rates of irritable bowel syndrome (IBS) are similar (10-20%) in most industrialized countries.[1–3] Limited available data suggest that IBS is also common in developing countries.[4–7] The prevalence of IBS was 24.4% in the first community-based survey using Rome-II criteria in rural Bangladesh.[8] IBS prevalence estimates vary depending on geographical locations and the diagnostic criteria used.[9–15] By using Rome-II criteria, authors reported a variable prevalence of IBS in national (12.1%)[16] urban (8.5%),[14] and suburban (5%)[12] surveys. Significant differences exist between urban and rural populations in Bangladesh with respect to the access to healthcare, socio- cultural, and environmental factors, which may theoretically affect epidemiological features.

Only 10-56% of adults with symptoms of IBS present for medical evaluation.[3,10,17] This may relate to cultural factors,[7] the presence and degree of pain and psychological disturbances,[18] and access to health care. Women with IBS consult physicians more than men do, although, such differences were not found in India,[19] Srilanka,[7] or in rural Bangladesh[8] (34.6 vs. 35.3%). Nevertheless, data on various aspects of IBS are lacking in the urban community of Bangladesh.

The present study aimed to find out the overall prevalence of IBS and healthcare-seeking patterns in an urban community of Bangladesh using the Rome-II definition. One of the purposes of this study was to arrive at a diagnosis without the help of investigations.

## MATERIALS AND METHODS

This observational study was conducted from the months of November 2004 to March 2005. A valid questionnaire was administered using home-based personal interviews to 1503 subjects aged 15 years and above in a defined area of Dhaka city of Bangladesh. Door-to-door surveys were carried out by physicians assisted by community healthcare workers.

### Questionnaire

The questionnaire was based on a previously published study[16] conducted abroad using internationally accepted Rome-II criteria for diagnosis. The Bengali translation of the questionnaire was validated by the method of forward and backward translation. For quality assurance and to evaluate the comprehensibility and appropriateness of the information, an initial survey with the questionnaire was done on 30 selected samples. That initial survey showed that the questions were easily understandable by the study population.

No laboratory tests or endoscopic examinations were done in the study due to lack of feasibility.

### Study definitions

For this study, “Rome-II” definition of IBS required abdominal pain and at least two or more of the three abdominal pain symptoms [Appendix-1] occurring more than 25% of the time in the preceding one-year.

### Statistical analysis

The data were processed for handling, and statistical analysis was performed with the computer-based Statistical Package for Social Science (SPSS) Programmers. Significance values were assessed during comparisons using the Chi-squared test with Yates' correction whenever necessary, and the level of significance was set at *P* ≤ 0.05.

## RESULTS

The mean age of the study population was 32.18±12.98 years with an age range of 15 to 97 years. The crude age- and gender-specific prevalence estimates and the prevalence of IBS subgroups have been described in Table 1. The overall, unadjusted estimate of IBS was 7.7 cases per 100 (*n* = 116) using the Rome-II study definition. The gender-specific prevalence was 8.6 cases per 100 (*n* = 67) in women, and 6.7 cases per 100 (*n* = 49) in men. IBS was 1.36 times more prevalent in females than in males (*P* > 0.05). The majority (*n* = 90; 77.58%) of the IBS cases were in the 15–44 years' age group [Table 1]. No other socio-demographic variable (occupation, financial status) was found to affect IBS prevalence estimates.

**Table 1:** Prevalence of IBS and IBS subgroups

**Variable**	**Male *n* (%)**	**Female *n* (%)**	**Total *n* (%)**
Prevalence	49 (6.7)	67 (8.6)	116 (7.7)
Age prevalence (years)			
15–24	16 (13.8)	20 (17.2)	36 (6.9)
25–34	13 (11.2)	16 (13.8)	29 (8.0)
35–44	7 (6.0)	18 (15.5)	25 (8.2)
45–54	7 (6.0)	9 (6.0)	16 (7.2)
55–64	5 (4.3)	2 (1.72)	7 (10.8)
> 65	1 (0.9)	2 (1.72)	3 (13.04)
IBS subgroups			
C-IBS	7 (36.8)	12 (40.0)	19 (16.4)
D-IBS	28 (48.3)	30 (51.7)	58 (50.0)
Neither C-IBS or D-IBS	12 (63.1)	18 (60.0)	30 (25.9)
Sometimes diarrhea			
sometimes constipation	2 (33.33)	7 (66.6)	9 (7.8)

### IBS subgroups

IBS subgroups summarized in Table 1 show that the predominant IBS subgroup was ‘diarrhea-predominant IBS,’ to which 58/116 (50.0%) belonged. Although the overall differences were not significant, all subgroups were found to be more prevalent in women.

### Prevalence of symptoms

The symptom responses of subjects summarized in Table 2 reveal that the association of abdominal pain with a change in stool frequency (100%, *n* = 116) and consistency (88.8%, *n* = 103) was quite common in the urban community. Colonic pain tended to be spastic in nature, *i.e*., the pain was relieved by defecation in 37.0% (*n* = 43) of the IBS cases. All IBS symptoms were more prevalent in women than in men and these differences were statistically significant (*P* < 0.000).

**Table 2:** Percentage of subjects having different IBS symptoms

**Symptoms**	**Male, *n* (%)**	**Female, *n* (%)**	***P* value**
Pain with more	45 (38.79)	60 (51.72)	0.000
bowel movement			
Pain with less	4 (3.4)	7 (6)	0.000
bowel movement			
Pain associated	37 (31.8)	49 (42.0)	0.000
with loose stools			
Pain associated	13 (11.2)	17 (14.7)	0.000
with hard stools			
> 3 bowel motions	9 (7.8)	20 (17.2)	0.000
per day			
< 3 bowel motions	1 (0.86)	5 (4.3)	0.000
per week			
Straining	4 (3.4)	10 (8.6)	0.000
Urgency	2 (1.7)	4 (2.4)	0.000
Incomplete	10 (9.6)	15 (12.9)	>0.05
evacuation			
Pain relieved by	13 (11.1)	30 (25.9)	0.000
defecation			
Mucus with stool	6 (5.1)	9 (7.7)	0.000
Bloating/	18 (15.5)	35 (30.2)	0.000
distension			

### Associated symptoms

The majority (84, 72.6%) of the IBS subjects reported having dyspeptic symptoms with heartburn in 84, of which were 30.17% were male and 42.24% were female. Frequent belching was seen in 78 (67.2%; 30.2% males and 37.06% females), nocturnal abdominal pain was seen in 38 (32.7%), and vomiting was seen in nine (7.7%) cases. All the dyspeptic symptoms like heartburn and frequent belching were found in higher frequency in females; these differences were statistically significant (*P* < 0.000).

### Healthcare-seeking pattern

Approximately 76 (65.51%) IBS subjects consulted a physician in the past one year with a higher rate of women consulters (68.6 vs. 61.2%; *P* >0.05). The consultation rate was found to increase with increasing age (*P* = 0.002) with heartburn and frequent belching being the two most common causes for consultation [Table 3]. No significant difference was noted in the prevalence of cardinal symptoms between consulters and nonconsulters except that the pain decreased with bowel movement for the consulters (*P* = 0.031). The main predictor for seeking healthcare was the presence of multiple dyspeptic symptoms. Only nine (7.7%) IBS subjects consulted healthcare professionals for the severity of their symptoms.

**Table 3:** Reason for consultation

**Symptoms**	**Total (%)**
Abdominal pain relieved by defecation	12 (10.3)
Abdominal pain with frequent motion	9 (8.53)
Abdominal pain with loose stools	11 (9.4)
Mucus with stool	4 (3.4)
Urgency	14 (12.0)
Sense of incomplete evacuation	7 (6.0)
Heartburn	47 (40.5)
Frequent passage of flatus	2 (1.7)
Frequent belching	43 (37.0)
Rumbling in abdomen	2 (1.7)
Multiple symptoms	65 (56.0)
None	3 (2.5)

## Discussion

The prevalence of IBS was found to be 7.7% using Rome-II criteria in this first urban community-based study. Prevalence was found to be 24.4% according to the same criteria, but only 8.5% when these criteria were strictly applied in the only rural community-based survey reported to date.[8] This low prevalence of IBS may be due to the use of the strict Rome-II criteria, which require symptoms to be chronic or recurrent for at least 25% of the time in the past one year. The prevalence estimate of this study is comparable to that found in studies conducted in western urban areas.[3,9,20] Variable prevalence of IBS was found in national (12.1%),[16] urban (8.5%),[14] and suburban (5.0%)[12] surveys using the Rome-II criteria. But only few of these studies compared the prevalence estimates of IBS among urban or rural population. Despite significant differences between urban and rural populations of Bangladesh with respect to psycho-socio-cultural and environmental factors, IBS prevalence was found to be similar when strict criteria (Rome II) were used for both these types of populations.

A greater prevalence of IBS symptoms in women than in men supports the findings of previous reports.[8,11,12,18] Like in other studies,[8,15] a downward trend of IBS prevalence with increasing age was also noted in the present data.

Abdominal pain associated with a change in stool frequency and/or consistency was present in all the IBS (*n* = 116) cases in this survey [Table 2]. This is consistent with the suggestion that abdominal pain is the dominant symptom of IBS.[10] Recurrent intestinal pain was also commonly experienced by IBS subjects in rural Bangladesh[8] and in western countries.[9–11] The data of the present study differ from that of Masud *et al*.[8] in the lower prevalence of altered stool passage, passage of mucus with the stool, and abdominal distension. On the other hand, altered frequency and consistency of stool associated with abdominal pain was found more frequently in this study than in the study reported by Masud *et al*.[8] Kapoor *et al*.[19] reported a higher prevalence of altered stool passage than of the other bowel symptoms in the Indian population.

All the IBS symptoms were found more prevalent in women than in men in the study. These data conform to the findings of other studies,[9,11] except for symptoms like loose stools and frequent motion. In the rural population, however, all the symptoms were found to be more prevalent in males, except for altered stool form and stool frequency.[8] These differences may be related to socio-cultural factors.

No investigation was done on the IBS subjects due to lack of feasibility. Also, Rome-II criteria had a sensitivity of 94% and specificity of 100%, and in the absence of any alarming symptoms, these criteria have a positive predictive value of approximately 98% with additional diagnostic tests having a yield of ≤ 2%.[21]

Whereas “diarrhea-predominant IBS” (*n* = 58) was the predominant IBS subgroup (50%) in the urban survey, only 0.8% cases had diarrhea-predominant IBS in the rural survey.[8] The reason for this high prevalence of diarrhea-predominant IBS may be due to different dietary habits and stressful life situations of the urban population. However, Thompson *et al*, reported almost equal prevalence of diarrhea-predominant and constipation-predominant IBS in a Canadian population according to Rome-II criteria.[16] All the subgroups were found to be more prevalent in females, except for the diarrhea-predominant type in this study. Talley *et al*.[20] reported almost equal prevalence of all the subgroups among males and females using Manning's criteria. Age and symptom patterns were not found to be significant discriminators for any of the subgroups, which was consistent with findings reported by Masud *et al*.[8] and Talley *et al*.[20]

The majority of IBS subjects (*n* = 76) had consulted a physician in the past one year, and there was a slightly higher rate of women consulters (68.6 *vs*. 61.2%). These findings are consistent with those of Heaton *et al*.[11] and Jones *et al*.[10] Talley *et al*.[20] however, did not see any differences between men and women in their healthcare-seeking behaviour. The rate of consultation was higher in urban IBS subjects than in rural people.[8] This may be due to a higher level of awareness and easier access to healthcare facilities for the urban population of Dhaka city. The rate of consultation was found to increase with increasing age in the present study, which was consistent with the findings of Jones *et al*.[10] but not with those of Masud *et al*.[8] and Heaton *et al*.[11] The majority (*n* = 62; 81.57%) of IBS subjects were found to consult for multiple symptoms in this study. Masud *et al*.[8] had reported that consultation rates declined with an increasing number of symptoms. In contrast to Talley's data,[20] the data from the present study conform to the study report of Heaton *et al*.[11] No significant difference was found in the colonic symptom patterns among consulters and nonconsulters except for pain decreasing with bowel movement; which was less prevalent in the nonconsulters. Dyspeptic symptoms were found to be less prevalent in the nonconsulters than in the consulters. Other than abdominal pain, multiple dyspeptic symptoms were the most important cause for consultation in this study [Table 3]; the next common cause was urgency. But Masud *et al*.[8] reported altered stool passage, a sense of incomplete evacuation, and the passage of mucus as predictor symptoms. Drossman *et al*.[17] reported that the presence of abdominal distension and incomplete evacuation were important reasons for consultation whereas Heaton *et al*.[11] reported pain as a strong predictor. From the present data, we could not explain why dyspeptic symptoms were found to be a strong predictor for seeking healthcare.

## CONCLUSION

This study demonstrated that the prevalence of IBS in the urban population of Bangladesh was similar to that reported by most other recent population-based studies done in other countries and also to that of the rural population when compared on the basis of strict Rome-II criteria. From the findings of the present study, we can conclude that IBS prevalence does not seem to be confounded by the factors which are likely to differ between western and non-western lifestyles, or between urban and rural lifestyles. A good number of IBS subjects seek healthcare for their dyspeptic symptoms with increasing age, and the numbers of dyspeptic symptoms were found to be important discriminators to seek consultation. We still have a poor understanding of the triggers for consultation among these patients. More research is required and preferably with psycho-social assessment and some appropriate investigations to find out the exact prevalence of IBS in urban people and to clarify patients' reasons for seeking medical advice.

## Appendix I

**Table:**

Revised Rome Criteria (Rome II)
*At least 12 weeks, which need not be consecutive, in the preceding 12 months of abdominal discomfort or pain that has 2 of 3 features: Relieved with defecation; and/or Onset associated with a change in frequency of stool; and/or Onset associated with a change in form (appearance) of stool.
*Symptoms that cumulatively support the diagnosis of IBS: Abnormal stool frequency (for research purposes, “abnormal” may be defined as greater than 3 bowel movements per day and less than 3 bowel movements per week); Abnormal stool form (lumpy/hard or loose/watery stool); Abnormal stool passage (straining, urgency, or feeling of incomplete evacuation); Passage of mucus; Bloating or feeling of abdominal distension.
